# Non-ischemic Cardiomyopathy Patients Derive Superior Mortality Benefit from Cardiac Resynchronization Therapy

**Published:** 2007-10-22

**Authors:** Jonathan Rosman, Sandeep Dhillon, Alexander Mayer, Sam Hanon, Schweitzer Paul

**Affiliations:** Beth Israel Medical Center, University Hospital and Manhattan Campus for the Albert Einstein College of Medicine New York, New York

**Keywords:** Cardiac Resynchronization Therapy, Bi-ventricular pacemaker, Cardiomyopathy

## Abstract

**Background:**

Cardiac Resynchronization Therapy (CRT) is indicated for the treatment of advanced heart failure with severe systolic dysfunction and intraventricular conduction delay. Patient selection for this technology is vital, though it remains unclear which patients benefit most from CRT. We tested the hypothesis that patients with non-ischemic cardiomyopathy have a superior mortality benefit from CRT than ischemic cardiomyopathy patients.

**Methods:**

We evaluated 95 CRT patients to determine which factors predict mortality.

**Results:**

Patients with non-ischemic cardiomyopathy had a significantly better prognosis than patients with ischemic cardiomyopathy.

**Conclusion:**

Larger prospective studies can substantiate this finding and better delineate which patients benefit most from CRT.

## Introduction

Patients with heart failure may remain symptomatic despite the use of pharmacological treatment. Many of these patients have wide QRS intervals leading to asynchronous contraction of the ventricles. Cardiac resynchronization therapy (CRT) improves cardiac hemodynamics, reduces clinical symptoms and decreases morbidity and mortality in these patients [1-6].

The criteria for CRT placement have changed since the advent of biventricular pacemakers. While many patients with heart failure benefit from CRT, it is still unclear which patients are ideal candidates for biventricular pacing. We sought to identify which patients benefit most from CRT.

## Methods

Ninety five consecutive patients who had a bi-ventricular pacemaker inserted at Beth Israel Medical Center between 7/02 and 11/05 were evaluated. All patient characteristics and clinical measures assessed prior to pacemaker implantation were analyzed to determine their impact on patient survival: age, gender, etiology of cardiomyopathy, bundle type, ejection fraction, left ventricular end diastolic dimension (LVEDD), and QRS complex duration. Patients whose EKG was paced prior to biventricular pacemaker insertion were not included in analysis for bundle type and QRS duration.

Univariate log-rank tests were initially used to investigate possible relationships (at p ≤ .10) between survival and each patient characteristic.  Subsequent multivariate analysis of potential risk factors for survival controlling for age was performed using Cox proportional hazards analyses. The partial likelihood ratio test was used to assess parameter significance at p ≤ .05. The study was approved by the Beth Israel Medical Center Institutional Review Board.

## Results

Baseline characteristics are shown in Table 1.

Age at time of pacemaker implantation varied from 33 to 89 years, with a median of 70 years.  Mean duration of follow-up was 1.6 years.   Twenty one patients (22% of males and 23% of females) died prior to study end-date, with death occurring 0.1 to 2 years (median 0.5 years) after pacemaker implantation.  To date, the duration of survival for the other 74 patients ranges from 0.7 to 3.9 years (median 1.7 years).

Initial log-rank tests revealed that age and etiology of heart disease were strongly related to survival (Table 2).

31% of patients with ischemic cardiomyopathy reached the endpoint of death versus 13% of patient with non-ischemic cardiomyopathy (p<.05; hazard ratio 3.29). Survival was not associated with gender, ejection fraction, QRS length, bundle type, or LVEDD. In addition, there was no medication associated with decreased mortality.

## Discussion

The present study investigated whether any pre-CRT baseline characteristics were associated with a better prognosis than other baseline characteristics. Our data demonstrated that patients with non-ischemic cardiomyopathy had decreased mortality with CRT compared to patients with ischemic cardiomyopathy. Our finding are consistent with prior studies [1]. In CARE-HF study 54% of patients with ischemic cardiomyopathy reached the primary endpoint of death or major cardiac event versus 39% of patients with non-ischemic cardiomyopathy (p<.0001).

There are two main limitations of our study. Our data is retrospective and we don't have all baseline characteristics on all 95 patients. In addition, we were unable to evaluate for any subjective functional improvements with CRT in the different groups.

The present study suggested that non-ischemic cardiomyopathy patients with CRT have a better prognosis than ischemic cardiomyopathy patients with CRT. Prospective studies, with larger samples specifically evaluating etiology of cardiomyopathy in CRT patients are needed to substantiate this finding.

## Figures and Tables

**Table 1:**
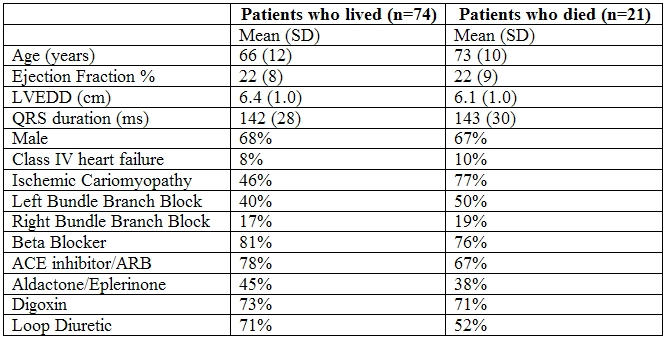


LVEDD= left ventricular end diastolic diameter; ARB= angiotensin II receptor blockers

**Table 2:**
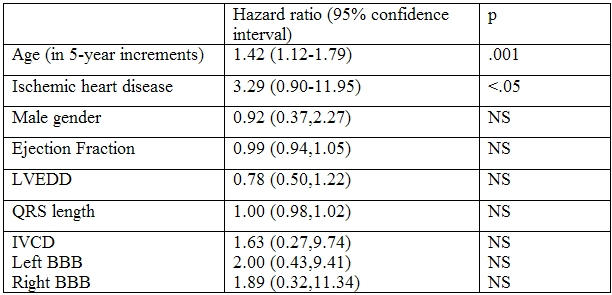


LVEDD= left ventricular end diastolic diameter; IVCD= intraventricular conduction defect; BBB= bundle branch block

## References

[R1] Cleland JG, Daubert JC, Erdmann E (2005). Cardiac Cardiac Resynchronization-Heart Failure (CARE-HF) study investigators. The effect of cardiac resynchronization on morbidity and mortality in heart failure. N Engl J Med.

[R2] Abraham WT, Fisher WG, Smith AL (2002). Multicenter InSync Randomized Clinical Evaluation. Cardiac resynchronization in chronic heart failure. N Engl J Med.

[R3] Breithardt OA, Stellbrink C, Franke A (2002). Pacing therapies for congestive heart failure study group. Guidant congestive heart failure research group. Acute effects of cardiac resynchronization therapy on left ventricular Doppler indices in patients with congestive heart failure. Am Heart J.

[R4] Yu CM, Chau E, Sanderson JE (2002). Tissue doppler echocardiographic evidence of reverse remodeling and improved synchronicity by simultaneously delaying regional contraction after biventricular pacing therapy in heart failure. Circulation.

[R5] Young JB, Abraham WT, Smith AL (2003). Multicenter InSync ICD Randomized Clinical Evaluation (MIRACLE ICD) Trial Investigators. Combined cardiac resynchronization and implantable cardioversion defibrillation in advanced chronic heart failure. JAMA.

[R6] Cazeau S, Leclercq C, Lavergne T (2001). Effects of multisite biventricular pacing in patients with heart failure and intraventricular conduction delay. N Engl J Med.

